# Regional Regulation of Purkinje Cell Dendritic Spines by Integrins and Eph/Ephrins

**DOI:** 10.1371/journal.pone.0158558

**Published:** 2016-08-12

**Authors:** Tristan G. Heintz, Richard Eva, James W. Fawcett

**Affiliations:** John van Geest Centre for Brain Repair, University of Cambridge, Robinson Way, Cambridge CB2 0PY, United Kingdom; University of Nebraska Medical Center, UNITED STATES

## Abstract

Climbing fibres and parallel fibres compete for dendritic space on Purkinje cells in the cerebellum. Normally, climbing fibres populate the proximal dendrites, where they suppress the multiple small spines typical of parallel fibres, leading to their replacement by the few large spines that contact climbing fibres. Previous work has shown that ephrins acting via EphA4 are a signal for this change in spine type and density. We have used an *in vitro* culture model in which to investigate the ephrin effect on Purkinje cell dendritic spines and the role of integrins in these changes. We found that integrins α3, α5 and β4 are present in many of the dendritic spines of cultured Purkinje cells. pFAK, the main downstream signalling molecule from integrins, has a similar distribution, although the intenstity of pFAK staining and the percentage of pFAK+ spines was consistently higher in the proximal dendrites. Activating integrins with Mg2+ led to an increase in the intensity of pFAK staining and an increase in the proportion of pFAK+ spines in both the proximal and distal dendrites, but no change in spine length, density or morphology. Blocking integrin binding with an RGD-containing peptide led to a reduction in spine length, with more stubby spines on both proximal and distal dendrites. Treatment of the cultures with ephrinA3-Fc chimera suppressed dendritic spines specifically on the proximal dendrites and there was also a decrease of pFAK in spines on this domain. This effect was blocked by simultaneous activation of integrins with Mn2+. We conclude that Eph/ephrin signaling regulates proximal dendritic spines in Purkinje cells by inactivating integrin downstream signalling.

## Introduction

The dendrites of cerebellar Purkinje cells have two zones. The proximal zone has a few clusters of spines, each cluster being innervated by a climbing fibre varicosity. The more distal branches of the dendrites have a much higher spine density, each spine corresponding to a parallel fibre input. The low spine density in the proximal dendrites is a result of the presence of climbing fibre connections which compete out parallel fibres. This competition depends on electrical activity, and if climbing fibres are removed or silenced the proximal region will now become populated by parallel fibres. As the parallel fibres take over, the spine density increases and the spines now have the appearance of those normally seen on the distal dendrites. The parallel fibre synapses are stabilised by the presence of GluRδ2 receptors, which may act as adhesion molecules [1, 2]. A mechanism for the action of climbing fibres on proximal dendrite spine density has recently been suggested; Cesa et al. (2011)[3] showed that climbing fibres repress proximal spines through a mechanism involving Eph/ephrin signalling. Blocking both ephrinA and ephrinB effects by infusion of EphA4-Fc led rapidly to a proliferation of spines in the proximal dendrite, while infusion of ephrin A2 or ephrinB1 suppressed the proliferation of proximal spines that occurs if the climbing fibre effect is removed by action potential block. Moreover animals lacking ephB1, B2 and B3 had a high spine density in the proximal dendrite despite the presence of climbing fibres, suggesting that one or more of these molecules is the receptor on Purkinje cells for ephrin released from the climbing fibres. Purkinje cells express multiple eph receptors, including A4, A5, A7 and B2 and also the ephrins B1, B3, B2, A4, A5, while the inferior olive, the nucleus of origin of the climbing fibres, expresses ephA4 and ephrinB3 [4–8]. The mechanism by which eph/ephrin signalling might affect spines has been reported to involve RhoA signalling, slingshot signalling, a pathway via CDK5, and an astrocyte-mediated pathway via integrins [9–14]. Spine formation, stability, motility and morphology is affected by multiple factors, one of which is the action of integrins [15–19]. Integrins are heterodimers of an alpha and beta subunit, and several types of heterodimer are present on the postsynaptic side of most synapses. The different heterodimers have different actions, binding to different ligands and linking to different intracellular signalling pathways. In synaptic biology beta3 integrins are particularly associated with synaptic scaling and regulation of AMPA receptors, alpha5 in spine formation, alpha3 in spine stability, beta1 in spine elongation and long-term potentiation. [20–23] and reviewed in [17, 24]. The identity of the integrin ligands in the synaptic space is still controversial, but application of the RGD peptide, which blocks the RGD motif present on fibronectin and several other ECM glycoproteins evokes changes in spine shape and stability [23]. Integrins signal mainly through the intracellular tail of the beta integrin, via two kinases focal adhesion kinase (FAK) and integrin-linked kinase (ILK). In the hippocampus activation of the EphA4 receptor affects the structure of dendritic spines by influencing integrin β1 actions [25], and in the cerebellum alphaVbeta3 integrin is present in Purkinje cell dendritic spines and is involved in LTP [26]. In light of these studies, we decided to investigate whether an integrin mechanism might mediate the effects of Eph/ephrin signalling from climbing fibres as they control the spines on the proximal dendrites of Purkinje cells. We have used *in vitro* models to study the effects of climbing fibres on Purkinje cell spines and also direct eph/ephrin effects on Purkinje cell spines and we have modulated integrin function in these models. We demonstrate an ephrin effect on integrin activation in spines, and effects on spine shape and density after modulating the function of integrins.

## Materials and Methods

### Coverslips

Coverslip preparation is critical to obtain good cell adhesion and achieve long lasting cell culture. 13 mm glass coverslips were carefully washed with 1M nitric acid for at least 3 hours, thoroughly rinsed at least three times with distilled water, placed independently on a filter membrane and then autoclaved. When finally dried, coverslips were coated with a solution of PDL (MW > 300 000) at 1mg / ml (Sigma) and placed in a 37°C incubator. Coverslips were then rinsed 3 times in dH20 and dried out before any plating of cells.

### Mixed Cerebellar Culture

E18 embryos were removed by cesarean-section from a terminally CO2-anesthetized pregnant Sprague-Dawley rat. The embryos were sacrificed by decapitation, and placed in ice-cold HBSS-CMF dissecting medium. After removing the brain from the cranial cavity, the cerebellum was isolated and kept in ice-cold Ca2+/ Mg2+-free Hank’s balanced salt solution (Gibco 21250). The cerebella were then digested in 2 ml of HBSS containing 0.1% trypsin at 37°C for 10 minutes. The digested cerebella were then rinsed twice with 8 ml of plating media and centrifuged for 3 minutes at 1000 rpm. They were then gently triturated with 3 polished Pasteur pipette of decreased diameter until the solution became opaque. Then, after settling down for 1 minutes, the cell suspension was collected and placed into a 15 ml falcon tube and cell density was adjusted to 5x 106 cells / ml in plating medium. A drop of 50 μl of the cell suspension was then plated on the glass coverslips of the central 8 wells of a 24 well plate, giving a final density of approximately 2.5x 106 cells per coverslips. After at least 3–4 hours of incubation in a 7% CO2 incubator, 900μl of serum-free culture media was added to each well giving a final concentration of 1% of serum in each well. The cells were then maintained in the incubator and fed once a week by replacing half of the medium with a fresh medium deprived of any serum but supplemented with bovine serum albumin (100 mg/ml; A3156, Sigma) and, in the case of pure neuronal condition experiments, with the glial proliferation inhibitor cytosine arabinoside (4 mM), (Tabata 2000; Furuya 1998). Experiments were conducted in accordance with the UK Animals (Scientific Procedure) Act, 1986, under a Home Office project licence to the University of Cambridge.

### Synaptoneurosome preparation

The method used for synaptoneurosome purification was that of [27]; please see this paper for details. In brief, synaptoneurosomes were prepared from pooled cerebellar cultures at DIV21 in homogenization solution, centrifuged at 1,000xg for 10 min at 4°C, and the pellet resuspended in 40% Optiprep (Sigma-Aldrich). This was spun at 16,500xg for 30 min at 4°C through an Optiprep step gradient. (35%, 25%, 15%, 12.5% and 9%). Fractions located at the two top interfaces were removed, pelleted at 20,000xg for 10 min and resuspended and loaded on a Percoll (Sigma-Aldrich) 25%, 20%, 16%,14% gradient. These were centrifuged 32,400xg for 20 min at 4°C. The synaptoneurosome fraction at the top of the 20% step (named 1P4) was removed pelleted by centrifugation at 20,000xg and resuspended for analysis.

#### Antibodies and reagents

Antibodies used in Purkinje cell experiment are: mouse monoclonal anti Calbindin (Swant 1/2000), guinea pig polyclonal anti calbindin (Synaptic System), chicken polyclonal anti calbindin (Swant 1/2000), rabbit polyclonal anti integrin β1 (Chemicon AB1952 1/500), rabbit polyclonal anti integrin β3 (Chemicon 1/500), rabbit polyclonal anti integrin β4 (Chemicon 1/500), rabbit polyclonal anti integrin α3 extracellular domain Ralph3.2 (Santa Cruz), rabbit polyclonal anti integrin α3 cytoplasmic domain (Chemicon AB1920 1/500), rabbit polyclonal anti integrin α5 extracellular domain (Chemicon AB1921 1/500), rabbit polyclonal anti integrin α5 cytoplasmic domain (Chemicon AB1928 1/500), rabbit polyclonal anti integrin αV (Chemicon), mouse monoclonal anti PSD95 (Affinity Bioreagents MA1-046, 1/500), rabbit polyclonal anti VGluT1 (Synaptic System), guinea pig polyclonal anti VGluT2 (Synaptic System), rabbit polyclonal anti pFAK (Santa Sruz), MAP2, Alexa fluor-conjugated secondary antibodies series were all purchased from Invitrogen and used at a dilution of 1/1000.

Manganese MnCl2, 4H2O, was acquired from Sigma and was used at a working concentration of 500 μM, corresponding to the concentration that provides a full activation of integrins. EphrinA3 was used at 10 μg / ml and purchased from R&D. Application of ephrin required preincubation with an Fc fragment to create an ephrinA3-Fc complex. 2.1.2.3 Reagents for cell culture Culture of dissociated Purkinje cells were prepared by dissecting cerebellum in a dissecting medium prepared with Hank's buffer salt solution (HBSS) Ca2+ and Mg2+ free, (HBSS-CMF, Gibco) supplemented with HEPES and kept at 4°C. Cells were then plated on coverslips in a plating medium (100 μl per well) containing a solution of DMEM / F12 (Gibco) supplemented with 10% FCS. After 3 hours of incubation in plating medium, cells were supplemented with a serum-free neuronal medium (900 μl) such as each well contained 1% of serum. The composition of the neuronal medium was as follow: DMEM / F12 (Gibco 31330–038), B27 (Gibco 17504044, 1/100), N2 (Invitrogen 17502048 1/100), Glutamax (Invitrogen 35050038 1/100), T3 hormone 0.5 ng/ml (Sigma T6397), BSA (100mg/ml, Sigma A3156) and a glial proliferation inhibitor: cytosine arabinoside AraC (4 mM, Sigma C1768).

### Immunofluorescence

Cell cultures were fixed in 2% PSF at RT for 10 minutes, rinsed three times in 1x PBS and blocked for 45 minutes at RT in a solution containing 1x PBS, 2% semi-skimmed milk (powder), and 0.3% Triton-X100. For integrin staining the cells were fixed with Methanol at −20°C for 5 minutes instead of PFA in order to preserve the integrity of the integrin epitope. After the blocking step, cells were rinsed three times for 10 minutes in PBS and either incubated overnight at 4°C with the primary antibodies diluted in blocking solution or incubated at room temperature for 1 hour. After incubation of the primary antibody, cells were washed three times in 1x PBS and finally incubated for 1 hour at room temperature in blocking solution containing the conjugated secondary antibodies, and in the case of integrin staining with biotinylated secondary antibodies. In this case the protocol requires an extra step consisting of washing the excess of biotinylated secondary antibody with PBS 3 times and finally incubating the cells streptavidin-conjugated antibody for another hour. Finally, cells were washed again in 1x PBS and mounted using Prolong Gold antifade reagent (Invitrogen) and let dry for 24 hours at room temperature (RT) before visualization.

### Image analysis and quantitative immunofluorescence

Immunolabeled cells were then visualized using a Leica CTR 6000B inverted confocal microscope coupled to a Leica DFL 350-FX camera and using the same microscope parameters, of laser intensity, gain and offset, for all the cells. The quantification of pFAK staining was performed in ImageJ by selecting an area of 16 pixels (4x4), image resolution 1024x1024, on top of every visible dendritic spine and then measuring the intensity of immunostaining in the corresponding channel in that area. The selection was displaced sequentially and manually from on spine to another and the same method was strictly applied to every measured cells in each conditions. The quantification of dendritic spines was performed by post-processing confocal z-stack images using ImageJ. Cells were immunostained against Calbindin 28K in order to visualize the morphology of Purkinje cells and their dendritic spines. The volume of z-stack was determined for each cell in order to capture the entire dendritic arborization. By moving through the z-stack spines can be identified more clearly that on a single picture. The length of dendritic spines was determined by tracing a segmented line from the base of the spine (the border of the dendritic shaft) to its apex. For each spine, the position in the z-dimension was carefully chosen so that the spines was optimally visualized. Simultaneously, the number of spines per visible segment was measured and provided an index of spine density (in spines per 10μm). Additionally, each spines was categorized by their morphology in one of the four groups proposed by Lee et al. [28]. An illustration of spines and their categorisation is shown in the figures. This method does not give the precision that could be achieved by electron microscopy, and it is not possible to quantify reliably spines that point vertically towards or away from the point of view.

### Statistical Analysis

All experiments in this section were conducted in blind conditions: the name of the different treatment groups were hidden during both the imaging and the quantification. The names of the different groups was only revealed after data analysis was completed. Statistical analysis was performed using the software GraphPad Prism v5.2 and the data files were organized using Microsoft Excel prior to analysis. All data analysis performed in sections 3.2 and 4.2 were analysed using a 2-way ANOVA followed by pairewised comparison. Each experiments was repeated 4 times. This number was used as the n parameter in the ANOVA. The results obtained are regrouped in Table 1 and Table 2 which summarise the p-value of each test).

**Table 1:** Effect of integrin and Eph activation on the number and the intensity of pFAK- positive spines in Purkinje cells.

		Control	Mn^2+^ 500μM	EphrinA3	EphrinA3/Mn^2+^
**Percentage of**	Proximal	23.38±3.1	50.45±4.3	7.14±4.5	30.7±10.1
**pFak^+^ spines**	Distal	8.87±2.3	31.57±5.7	6.35±2.4	26.2±7.2
**Intensity of**	Proximal	1.00±0.7	1.83±1.07	0.55±0.38	1.07±0.48
**pFak^+^ spines**	Distal	0.67±0.5	1.15±0.7	0.61±0.31	1.28±1.19

**Table 2:** Effect of integrin activation on length and density of spines in Purkinje cells.

	Control	Mn^2+^ 500μM	EphrinA3	EphrinA3/Mn^2+^	RGD
**Spine length**					
**(μm)** Proximal	1.03±0.35	0.99±0.31	0.83±0.36	0.98±0.49	0.74±0.37
Distal	1.01±0.24	0.91±0.27	0.99±0.32	0.93±0.28	0.68±0.29
**Spine density**					
**(sp/μm)** Proximal	2.07±0.42	2.46±0.43	0.65±0.18	1.64±0.34	1.92±0.29
Distal	2.93±0.88	3.09±0.10	2.80±0.61	2.71±0.41	2.75±0.38

## Results

### 1. Expression of integrins in Purkinje cells in vitro

**Fig 1:**
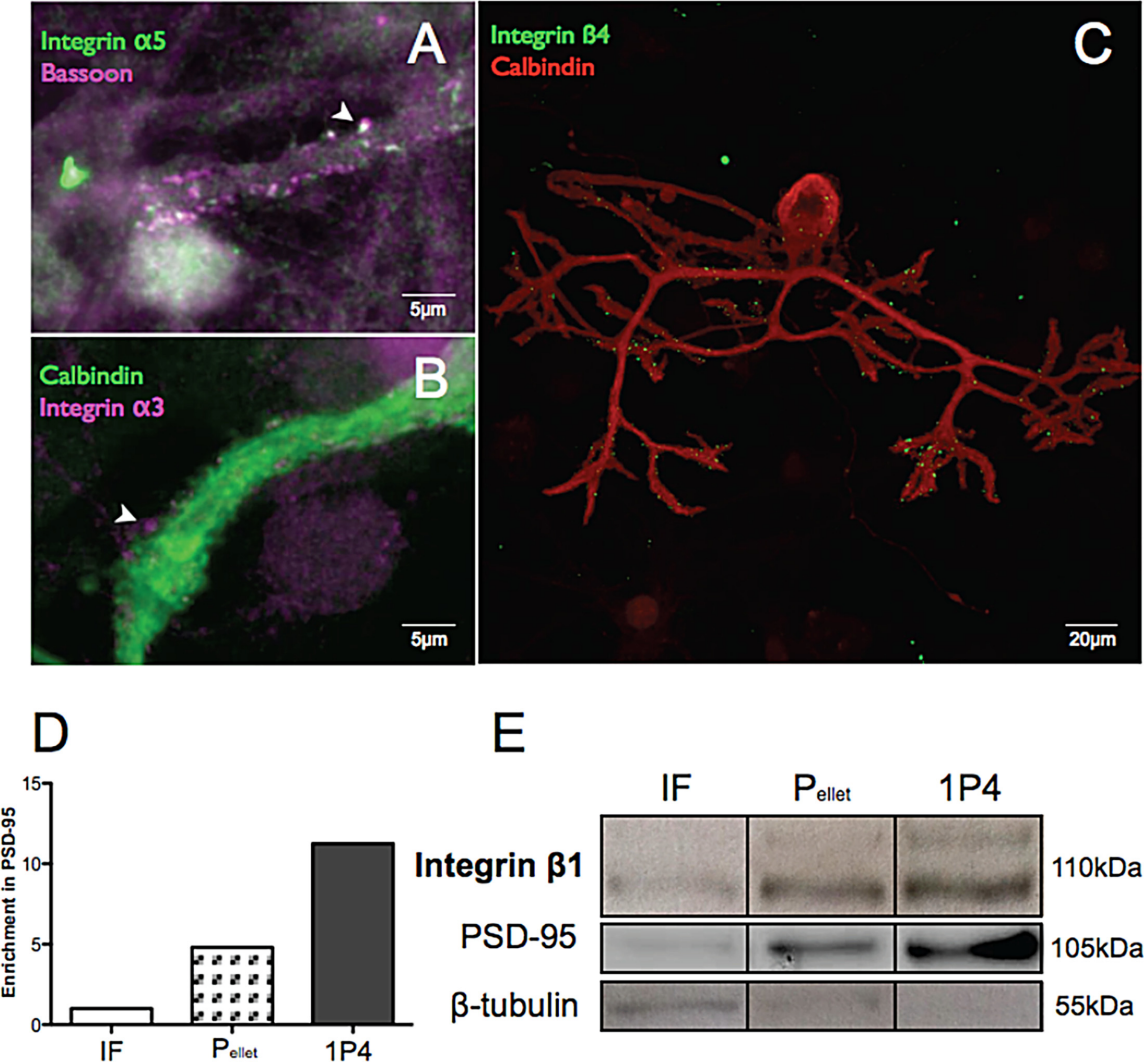
Presence of integrins in dendritic spines of Purkinje cells. Immunostaining against integrin α3 and α5 in Purkinje cells in culture (A-C) showing that integrins are expressed on the dendritic tree and in synapses where they are contiguous with the presynaptic marker bassoon (A, arrowhead). The presence of integrins in dendritic spines was further demonstrated by subcellular fractionation (E). IF = initial homogenate, P = spun down pellet, IP4 = synaptoneurosome fraction. β1 integrin was concentrated in the 1P4 fraction, which was also enriched with PSD-95 (D-E).

We first investigated the expression and distribution of integrins in our mixed cerebellar culture model, using immunofluorescence and subcellular fractionation followed by western blotting. Integrin immunofluorescence is limited by the availability of effective antibodies; we tested various antibodies in our culture model and obtained reproducible and specific stains using antibodies against α3, α5 and β4 two of which have previously been used for Purkinje cell staining [26]. We stained mixed cerebellar cultures at 7, 14 and 21 days in vitro using these antibodies, identifying Purkinje cells with an antibody to Calbindin 28k. There was no integrin staining at DIV 7, but at DIV21, mature Purkinje cells were positive for both α3 and α5 integrin. α 3 puncta were present on both soma, dendritic shaft and on a subset of spines but with no particular pattern of distribution (Fig 1B). Similarly, staining for integrin α5 was scattered homogeneously over the entire cell, but was prominent on dendritic spines identified by proximity to puncta of bassoon staining (Fig 1A). Integrin β4 staining was also seen on a subset of spines scattered over the dendrites (Fig 1C). Our results suggest that in conditions of mixed cerebellar culture deprived of climbing fibres, integrins are homogeneously distributed over the dendritic tree of Purkinje cells. In order to verify that integrins are present in the postsynaptic regions, we performed a subcellular fractionation of cerebellar cultures according to the method of [27], and found β1 integrin enriched in the synaptoneurosome fraction.

### 2. Activation of integrins induces an increase in pFAK

**Fig 2:**
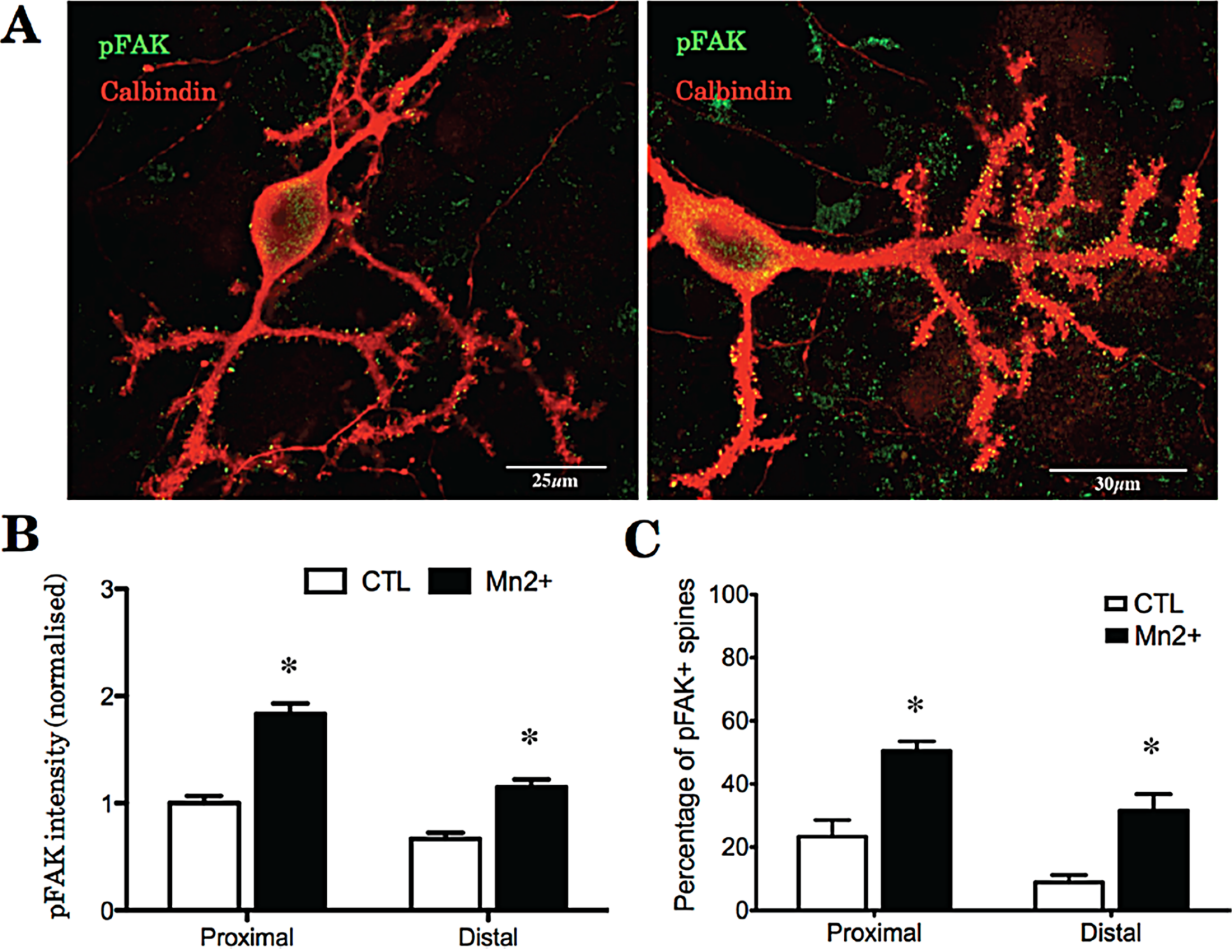
Manganese increases the number and the intensity of pFAK+ spines. Purkinje cells were treated with 500μM Mn2+ for 1 hour and then immunostained against pFAK (A left, control; A right, Mn2+). Before Mn2+ treatment the proportion of and intensity of pFAK spines is higher in the proximal segment. After Mn2+ treatment, both the number and the intensity of pFAK+ spines was increased throughout the dendrites.

**Fig 3:**
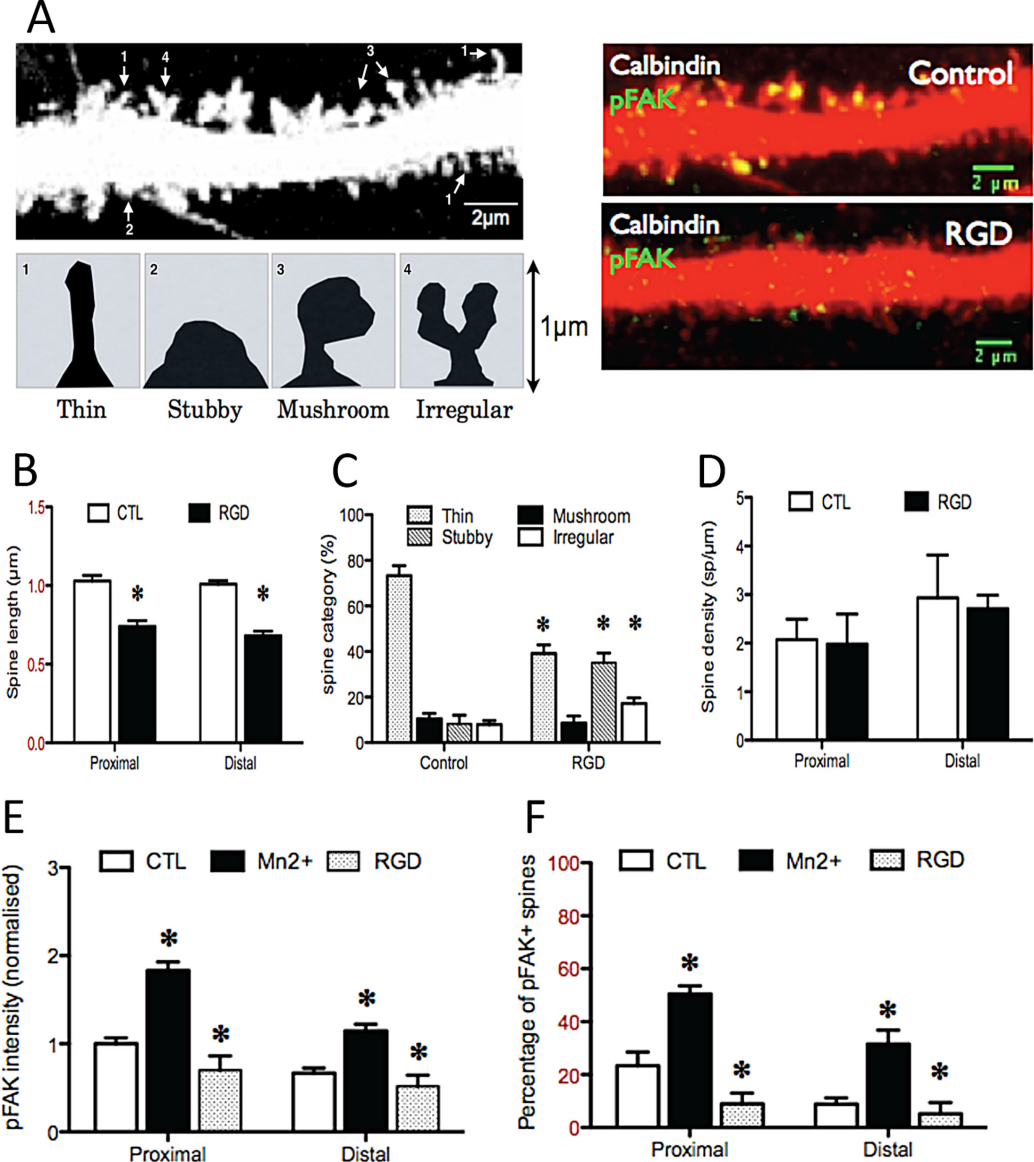
Effects of integrin manipulations on spine morphology, pFAK staining intensity and percentage of stained spines. An example of spine categorization is shown in A left: the picture shown to the right is enlarged and sharpened, and spine categories labelled. Treatment with RGD for 1h decreased pFAK intensity (A right green and E) in Purkinje cell dendritic spines (Calbindin, red, A) and the number and intensity of pFAK+ spines (F). RGD treatment also decreased spine length (B), with a more stubby phenotype rather than a thin one (C) but did not affect spine density on either the proximal or distal dendrites (D).

Activation of integrins in hippocampal and cortical neurons can lead to structural modifications of dendritic spines through a reorganization of the actin cytoskeleton. Since we found that integrins are expressed in many of the spines of Purkinje cells, we asked whether activation of integrins in these cells would also lead to changes in in the intensity and number of spines containing pFAK, the main downstream signalling molecule from integrins. We applied 500μM Mn2+ to Purkinje cell cultures at 21 days for one hour, and then fixed the cells for immunostaining against Calbindin, total FAK and pFAK. In order to verify integrin activation and signalling, and to give a further assessment of integrin distribution we quantified two parameters: the intensity of pFAK staining in individual spines of Purkinje cells, and the ratio of pFAK+/pFAK- spines per Purkinje cell (Fig 2), with separate counts for proximal (proximal to the first branch) and distal dendrites. In basal conditions 23.3% of proximal spines (±3.1, n = 3 repeated experiments) and 8.9% of distal spines (±2.3, n = 3) were positive for pFAK suggesting that in resting conditions, proximal dendrites contain more activated integrins than distal dendrites. After Mn2+ treatment, 50.4% of the spines on the proximal dendrite (±4.3, n = 3) and 31.5% of the spines on the distal dendrites (±5.7, n = 3) became positive for pFAK (Fig 2A and 2C; Tables 1 and 2). This increase was significant on both the proximal and the distal dendrites (p = <0.001) Mn2+ treatment therefore increased the number of dendritic spines containing activated integrins on both the proximal and the distal dendrites. We then quantified the intensity of pFAK after treatment with Mn2+. There was a substantial increase in the intensity of pFAK staining in dendritic spines treated with Mn2+, regardless of their dendritic compartment (Fig 2B). An example of the morphology of the spines and the effect of these interventions is shown at higher magnification in Fig 3. Taken together, these results demonstrate that many of the spines in Purkinje cells contain integrins and that treatment with Mn2+ leads to their activation and to phosphorylation of FAK.

### 3. Activation of integrins does not affect spine density, length or morphology in Purkinje cells

Since integrin signalling affects spine shape in cortical and hippocampal neurons, we looked at the effect of integrin activation on the density, length and morphology of dendritic spines. Cells were treated with 500μM of Mn2+ for 1h and then fixed for immunostaining. We labelled the cells for Calbindin to visualize Purkinje cells dendritic spines and used pFAK staining as a readout of integrin activation. We found that treatment with Mn2+ for 1h did not affect the length of spines in either the proximal (1.03± 0.35 μm Control and 0.89± 0.31 μm Mn2+, p = 0.6056) or the distal dendrites (0.85± 0.24 μm Control and 0.91± 0.27 μm Mn2+, p = 0.7498, ±SD (Tables 1 and 2). We then compared the density of spines on both proximal and distal dendrites before and after treatment. Similarly, there were no significant changes in spine density in either the proximal (2.07± 0.42 spines per μm Control and 2.46± 0.43 spines per μm Mn2+, p = 0.2453, ±SD) or the distal dendrites dendrites after Mn2+ treatment (2.93± 0.88 spines per μm in control conditions and 3.09± 0.1 spines per μm for Mn2+, p = 0.7383, ±SD, (Tables 1 and 2). These results suggest that the activation of integrins with Mn2+ does not by itself affect the size or density of Purkinje cell dendritic spines regardless of their dendritic localization.

### 4. Inhibition of integrin activity causes spine retraction

We show above that integrin activation by itself does not lead to significant changes in spine morphology, density or length in either the proximal or the distal dendrites: we therefore asked whether blocking integrin function would affect spines. Purkinje cell cultures were treated with an Arg-Gly-Asp (RGD)-containing peptide which acts as a competitive antagonist for RGD-targeted integrins, and which in previous work affected spine behaviour in hippocampal neurons [23]. We applied the RGD peptide for one hour in Purkinje cell culture and measured the length of dendritic spines and pFAK on both proximal and distal dendrites. Both the intensity of pFAK staining and the number of pFAK+ spines were decreased after RGD treatment (p = 0.02, n = 12 and p = 0.04, n = 12 cells) confirming the ability of this peptide to decrease integrin signalling (Fig 3A, 3E and 3F), also see last fig column RGD vs CTL. We then measured spine morphology on both proximal and distal dendrites. Inhibition of integrin function with an RGD peptide led to a reduction in spine length on both proximal and distal dendrites (p = 0.004, Fig 3B, Fig 5 column RGD), but had no effect on spine density (p = 0.3, n = 12 cells). Spine morphology was affected by RGD treatment, with a decrease in the proportion of Thin and Mushroom spines and an increase in the proportion of Stubby spines on both dendritic domains (Fig 3C Column 5 RGD). Taken together, these results suggest that integrin activity helps to maintain an elongated phenotype of spine on both proximal and distal dendrites, consistent with distribution of both integrins and pFAK on the dendritic tree.

### 5. EphrinA3-Fc induces a collapse of proximal but not distal spines

**Fig 4:**
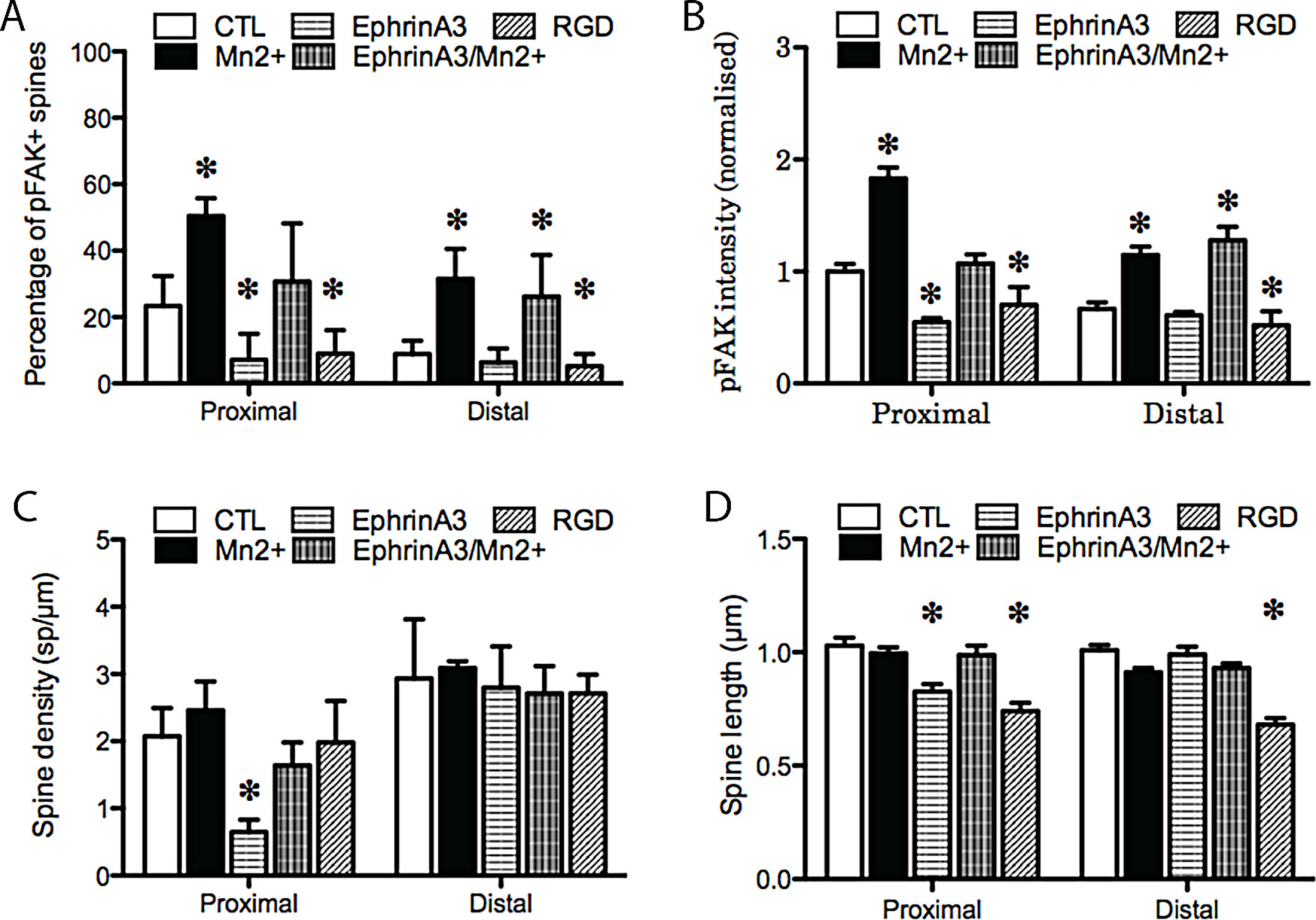
A. Quantitation of the effects of the various interactions on the percentage of spines positive for pFAK. B quantitation of effects on pFAK intensity. C quantitation of spine density. D quantitation of spine length. Treatment with 500μM of Mn2+ for 1 hour (B1-6) increased pFAK but had no effect on spine density. Treatment with EphrinA3 (C1-6) decreased the density of proximal spines and of integrin signaling, whereas combined treatment of Mn2+ and EphrinA3 (D1-6) restored both spine density and pFAK intensity to values closed to control. Finally, treatment with RGD peptide (E1-6) decreased pFAK intensity and spine length without affecting spine density.

**Fig 5:**
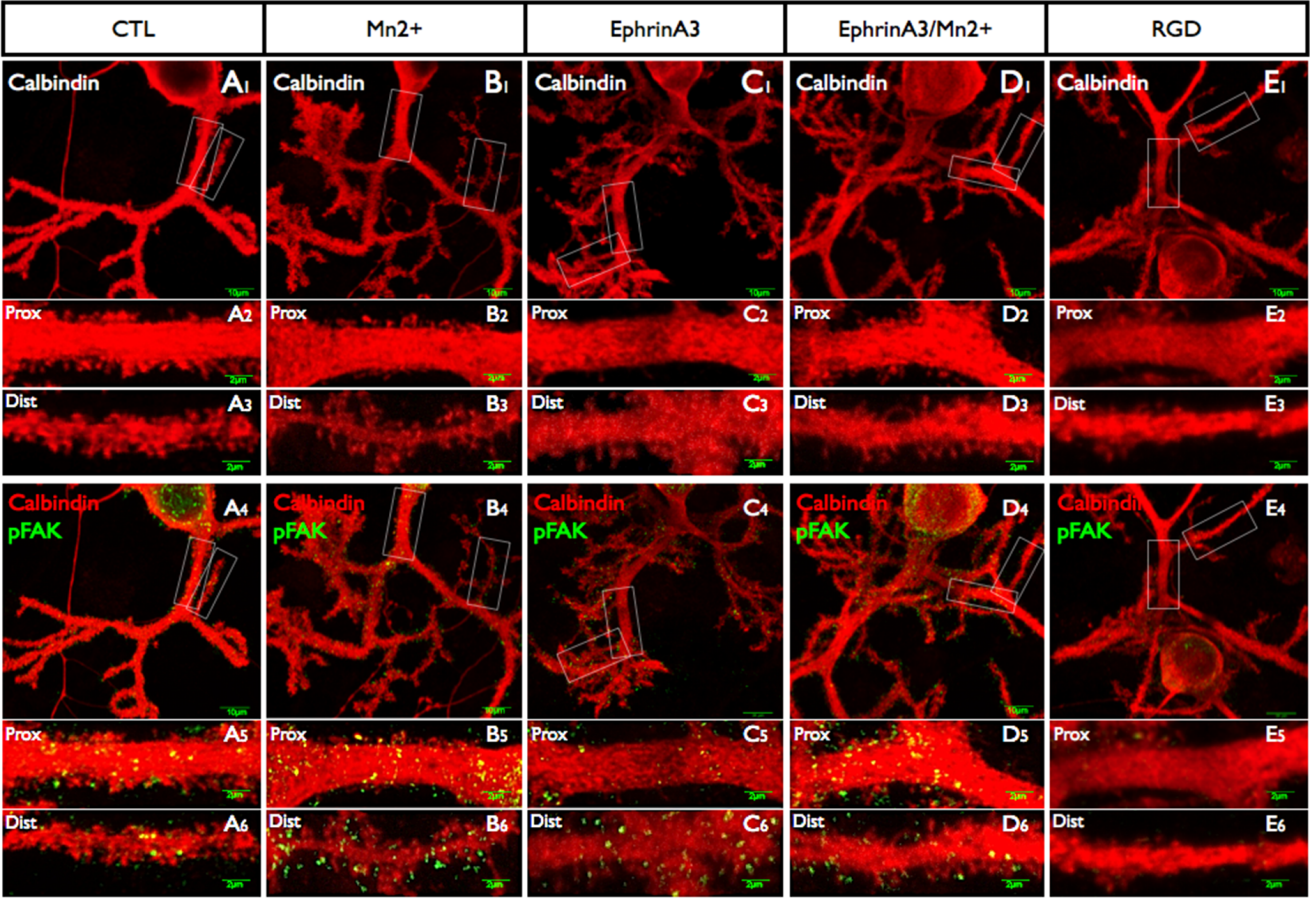
Changes in Purkinje cell dendrites imaged with calbindin and pFAK, with low power images of the dendritic tree and high power pictures of the proximal and distal dendrites. The various treatments applied to the cells include Mn2+, RGD peptide and ephrinA3. Quantitation of the spine morphology and pFAK is shown in Figs 2 and 4 and in Tables 1 and 2.

Purkinje cells express the EphA4 receptor at high level and are also reported to express Ephs A5, A7 and B2. They are therefore able to respond to ephrinA3 and other ephrins. We activated these receptors using ephrinA3-Fc for one hour in Purkinje cell cultures and quantified spine density on both proximal and distal dendrites. Under these conditions, activation of Eph receptors decreased the density of dendritic spines specifically on the proximal dendrites (0.65 spines per μm ± 0.18 versus 2.07 spines per μm ± 0.42 in controls p = <0.001, ±SD, Fig 4C) but not on the distal dendrites (2.8 spines per μm ± 0.61 versus 2.93 spines per μm ± 0.88 in controls, p = 0.8093, (Fig 4C- see also Fig 5 column Ephrin A3 vs. CTL). Additionally, treatment of Purkinje cells with ephrinA3-Fc made the spines on the distal dendrites appear more stubby, although the decrease in length was not significant (0.83μm ±0.36 and 1.03μm ±0.35 in controls, p = 0.4592) (Fig 4D, Fig 5 column Eprhin A3 vs. CTL)). These results indicate that Eph activation in a culture model deprived of climbing fibres suppresses proximal dendritic spines in Purkinje cells, similar to the effect of climbing fibres.

### 6. EphrinA3 inhibits integrin activity in proximal dendritic spines

We next investigated whether Eph activation causes a change in integrin signalling, assessed by measuring the percentage of stained spines and the staining intensity of pFAK using quantitative immunofluorescence. The application of ephrinA3 for one hour reduced the intensity of pFAK in spines on the proximal dendrites (from 1.00 ±0.68 to 0.55 ±0.38, intensity values normalized to control conditions on the proximal dendrites, p = <0.001, ±SD) but had no effect on spines on the distal domain (from 0.67 ±0.5 in control conditions to 0.61 ±0.31 after treatment with ephrinA3-Fc, p = 0.2)(Fig 4A and 4B, Fig 5 column Ephrin A3 lower panels vs. CTL). In addition to intensitye also measured the number of pFAK+ spines remaining after ephrin treatment. In accordance with the proximal collapse induced by ephrinA3, we found that the number of pFAK+ spines on the proximal domain was reduced by ephrin treatment (23.38% ±3.1 pFAK+ spines in control and 7.14% ±4.5 after treatment, p = 0.015) but was not affected on the distal dendrites (8.87% ±2.3 and 6.35% ±2.4 after treatment, p = 0.22) (Fig 4A, Fig 5 column Ephrin A3 bottom vs. CTL). Thus, both the number of pFAK+ spines and the intensity of pFAK staining in positive spines were reduced after ephrinA3 treatment.

### 7. Collapse of proximal spines by EphrinA3 is prevented by integrin activation

Based on the results above, our hypothesis is that ephrinA3 acts on EphA4 receptors to inhibit integrin signalling, which leads to the collapse of proximal but not distal dendritic spines. To support this hypothesis, we asked whether forced activation of integrins would prevent collapse of proximal spines by ephrinA3. Combined treatment with EphrinA3-Fc and Mn2+ did not affect the density or the morphology of spines on the distal dendrites (p = 0.16), and the presence of ephrinA3 did not reduce the number or intensity of pFAK spines compared to Mn2+ alone (26.2% ±7.2 of pFAK+ spines for the combination and 31.5% ±5.7 for Mn2+ alone on the distal dendrites, p = 0.18), suggesting that ephrins had little effect on spines of the distal dendrites (Fig 4A, 4B, 4C and 4D, Fig 5 column Mn2+ vs. EphrinA3/Mn2+). On the proximal dendrites the effect of Mn2+ moderated that of the ephrin. Co-treatment with ephrinA3 and Mn2+ increased the intensity of pFAK (p = <0.001), but the effects of Mn2+ on spine morphology were prevented: spine density (p = 0.16) and spine length (p = 0.92) were unaffected compared to control (Fig 4A, 4B, 4C and 4D, Fig 5 column Mn2+ vs EphrinA3/Mn2+). The data for these studies is present in the supporting information (S1 Table). Taken together, these results indicate that ephrinA3 induces a collapse of proximal dendritic spines that can be prevented by stimulating integrins. These results therefore strengthen the model of a regulation of proximal spines by ephrin action through an effect on integrin signaling.

## Discussion

### Summary of findings

We found that integrins α3, α5 and β4 are present in many of the dendritic spines of cultured Purkinje cells. pFAK, the main downstream signalling molecule from integrins, has a similar distribution, although the intensity of pFAK staining and the percentage of pFAK+ spines was consistently higher in the proximal dendrites. Activating integrins with Mg2+ led to an increase in the intensity of pFAK staining and an increase in the proportion of pFAK+ spines in both the proximal and distal dendrites, but no change in spine length, density or morphology. Blocking integrin binding with an RGD-containing peptide led to a reduction in spine length and more stubby spines on both proximal and distal dendrites. Treatment of the cultures with ephrinA3-Fc chimera suppressed dendritic spines specifically on the proximal dendrites and there was also a decrease of pFAK on this domain. This effect was blocked by simultaneous activation of integrins with Mn2+. We conclude that Eph/ephrin signaling regulates proximal dendritic spines in Purkinje cells by inactivating integrin downstream signaling.

### Eph/ephrin and integrin regulation of dendritic spines

Our work follows on from that of Cesa et al [3], in which it was shown that activation of the Eph receptors in Purkinje cells was responsible for much of the effect of climbing fibres in the suppression of spines on the proximal part of Purkinje cell dendrites. Our results support and augment these findings by suggesting that the effect of ephrins, probably acting via EphA4, is mediated by the inhibition of integrin signalling via pFAK. This is therefore a further example in which integrins are involved in the regulation of structural synaptic plasticity. A link between Eph/ephrin signalling and control of synapses via β1 integrin has been reported previously, with a similar regulation of dendritic spines by EphA4 and integrin β1 in hippocampal neurons [25]. There are many instances of synaptic and spine regulation by integrins, for instance via binding to ICAM-5, endostatin, SPARC, and the GluA2 AMPA receptor [29–31]. Integrin α3 and β3 in particular have a well-recognized role in synaptic stability and scaling [19, 20, 32]. Integrin effects are involved in various forms of LTP and other synaptic events, (reviewed in [16, 17, 19, 33]. Eph/ephrin interactions, both forward via Ephs and reverse via ephrins have many effects on synapse formation and plasticity (reviewed in [34, 35]. The particular interest in this paper is signalling from ephrinA’s, probably via EphA4. Here, several recent papers suggest that regulation of glutamate transporters and perisynaptic astrocytes are a key mechanism [36–38]. Our cultures certainly contained large numbers of astrocytes, so a mechanism of this type is credible for our findings. However, a direct effect on integrin signalling, as demonstrated in hippocampal neurons [25] is particularly likely in view of our demonstration that ephrinA3 application to neuronal cultures has a rapid effect on integrin activation.

### Why is the action of EphA4 restricted to the proximal dendrites?

In our experiments, and also in those of Cesa et al [3], the effects of Eph/ephrin interventions were restricted to the proximal dendrites. In the experiments of Cesa there were climbing fibres present, able to define the proximal dendritic domain by their specific innervation of this region. However in our experiments there were no climbing fibres, and the dendrites were not morphologically differentiated into a spine-poor proximal and spine-rich distal zone. Despite this, ephrinA3 had its effects, suppression of spines and reduction in pFAK specifically in the proximal dendritic region. Cesa et al also found that one week after the removal of climbing fibers, by which time the proximal dendrites had changed to a high synapse density, the actions on dendrites were still specific to the proximal domain. From our experiments it is not clear what the reasons for this might be. Purkinje cells express large amounts of EphA4, but this appears to be evenly spread over the entire dendritic tree [4, 39]. Also the three integrins that we stained for were evenly spread over the proximal and distal domains, and α3, α5 and αV integrins *in vivo* are reported to be spread evenly over the Purkinje cell dendrites [26, 40]. However, there is a suggestion of the mechanism from the distribution of pFAK in our Purkinje cells, which was consistently present in a higher proportion of spines and at a higher staining intensity in the proximal compared to the distal domain. It is possible, therefore, that some signalling event that impacts on integrin or FAK activation is concentrated in the proximal dendrites. Overall, the presence of domains within dendritic trees in which a particular input or molecule has a specific presence is commonplace within the nervous system, while the sort of continuous competition for synaptic space that occurs on Purkinje cells between climbing fibres and parallel fibres is less common.

Overall, our data suggest a mechanism for the action of climbing fibres to change the spines on the proximal dendrites of Purkinje cells from the many small spines typical of distal dendrites to the few large spines that contact climbing fibres. An ephrin is released either by the climbing fibres or the perisynaptic astrocytes. This acts on EphA4 on the Purkinje cell dendrites, which leads to a signalling process, restricted to the proximal dendrites, that inactivates integrins or FAK in the spines, leading to spine retraction.

## Supporting Information

S1 Table The excel file in supporting information provides raw data from the experiment examining the single and combined effects on ephrin A3, Mn2+ and RGD peptide which is illustrated in Fig 4. The data shows the size of dendritic spines in pixel numbers, followed by analysis of shape.
(XLSX)

## References

[1] Cesa R, Morando L, Strata P. Glutamate receptor delta2 subunit in activity-dependent heterologous synaptic competition. J Neurosci. 2003;23(6):2363–70. 12657696 10.1523/JNEUROSCI.23-06-02363.2003 PMC6742029

[2] Mandolesi G, Autuori E, Cesa R, Premoselli F, Cesare P, Strata P. GluRdelta2 expression in the mature cerebellum of hotfoot mice promotes parallel fiber synaptogenesis and axonal competition. PLoS ONE. 2009;4(4):e5243 10.1371/journal.pone.0005243 19370152 PMC2666267

[3] Cesa R, Premoselli F, Renna A, Ethell IM, Pasquale EB, Strata P. Eph receptors are involved in the activity-dependent synaptic wiring in the mouse cerebellar cortex. PLoS ONE. 2011;6(4):e19160 10.1371/journal.pone.0019160; PONE-D-10-05943 [pii]. 21559471 PMC3084771

[4] Greferath U, Canty AJ, Messenger J, Murphy M. Developmental expression of EphA4-tyrosine kinase receptor in the mouse brain and spinal cord. Mech Dev. 2002;119 Suppl 1:S231–8. . 14516691 10.1016/s0925-4773(03)00122-9

[5] Willson CA, Foster RD, Onifer SM, WHITTEMORE SR, Miranda JD. EphB3 receptor and ligand expression in the adult rat brain. J Mol Histol. 2006;37(8–9):369–80. 17103029 10.1007/s10735-006-9067-0

[6] Liebl DJ, Morris CJ, Henkemeyer M, Parada LF. mRNA expression of ephrins and Eph receptor tyrosine kinases in the neonatal and adult mouse central nervous system. J Neurosci Res. 2003;71(1):7–22. 12478610 10.1002/jnr.10457

[7] Saywell V, Cioni JM, Ango F. Developmental gene expression profile of axon guidance cues in Purkinje cells during cerebellar circuit formation. Cerebellum. 2014;13(3):307–17. 10.1007/s12311-014-0548-5 . 24550128

[8] Migani P, Bartlett C, Dunlop S, Beazley L, Rodger J. Ephrin-B2 immunoreactivity distribution in adult mouse brain. Brain Res. 2007;1182:60–72. 10.1016/j.brainres.2007.08.065 . 17945206

[9] Moeller ML, Shi Y, Reichardt LF, Ethell IM. EphB receptors regulate dendritic spine morphogenesis through the recruitment/phosphorylation of focal adhesion kinase and RhoA activation. J Biol Chem. 2006;281(3):1587–98. 16298995 10.1074/jbc.M511756200

[10] Carmona MA, Murai KK, Wang L, Roberts AJ, Pasquale EB. Glial ephrin-A3 regulates hippocampal dendritic spine morphology and glutamate transport. Proc Natl Acad Sci U S A. 2009;106(30):12524–9. 10.1073/pnas.0903328106 19592509 PMC2718351

[11] Murai KK, Pasquale EB. Eph receptors, ephrins, and synaptic function. Neuroscientist. 2004;10(4):304–14. 10.1177/1073858403262221 . 15271258

[12] Zhou L, Jones EV, Murai KK. EphA signaling promotes actin-based dendritic spine remodeling through slingshot phosphatase. J Biol Chem. 2012;287(12):9346–59. 10.1074/jbc.M111.302802 22282498 PMC3308827

[13] Fu WY, Chen Y, Sahin M, Zhao XS, Shi L, Bikoff JB, et al Cdk5 regulates EphA4-mediated dendritic spine retraction through an ephexin1-dependent mechanism. Nat Neurosci. 2007;10(1):67–76. 10.1038/nn1811 . 17143272

[14] Margolis SS, Salogiannis J, Lipton DM, Mandel-Brehm C, Wills ZP, Mardinly AR, et al EphB-mediated degradation of the RhoA GEF Ephexin5 relieves a developmental brake on excitatory synapse formation. Cell. 2010;143(3):442–55. 10.1016/j.cell.2010.09.038 21029865 PMC2967209

[15] Orlando c, Ster J, Gerber U, Fawcett JW, Raineteau O. Peridendritic chondroitin sulfate proteoglycans restrict structural plasticity in an integrin-dependent manner. J Neurosci. 2012;32:18009–17. 10.1523/JNEUROSCI.2406-12.2012 23238717 PMC6621736

[16] Senkov O, Andjus P, Radenovic L, Soriano E, Dityatev A. Neural ECM molecules in synaptic plasticity, learning, and memory. Prog Brain Res. 2014;214:53–80. 10.1016/B978-0-444-63486-3.00003-7 . 25410353

[17] Levy AD, Omar MH, Koleske AJ. Extracellular matrix control of dendritic spine and synapse structure and plasticity in adulthood. Front Neuroanat. 2014;8:116 10.3389/fnana.2014.00116 25368556 PMC4202714

[18] Vitureira N, Letellier M, Goda Y. Homeostatic synaptic plasticity: from single synapses to neural circuits. Curr Opin Neurobiol. 2012;22(3):516–21. 10.1016/j.conb.2011.09.006 21983330 PMC3378479

[19] McGeachie AB, Cingolani LA, Goda Y. Stabilising influence: integrins in regulation of synaptic plasticity. Neurosci Res. 2011;70(1):24–9. doi: S0168-0102(11)00046-0 [pii]; 10.1016/j.neures.2011.02.006 21352859 PMC3242036

[20] Kerrisk ME, Greer CA, Koleske AJ. Integrin alpha3 is required for late postnatal stability of dendrite arbors, dendritic spines and synapses, and mouse behavior. J Neurosci. 2013;33(16):6742–52. 10.1523/JNEUROSCI.0528-13.2013 23595732 PMC3711182

[21] Warren MS, Bradley WD, Gourley SL, Lin YC, Simpson MA, Reichardt LF, et al Integrin beta1 signals through Arg to regulate postnatal dendritic arborization, synapse density, and behavior. J Neurosci. 2012;32(8):2824–34. 10.1523/JNEUROSCI.3942-11.2012 22357865 PMC3313657

[22] Pozo K, Cingolani LA, Bassani S, Laurent F, Passafaro M, Goda Y. beta3 integrin interacts directly with GluA2 AMPA receptor subunit and regulates AMPA receptor expression in hippocampal neurons. Proc Natl Acad Sci U S A. 2012;109(4):1323–8. 10.1073/pnas.1113736109 22232691 PMC3268285

[23] Shi Y, Ethell IM. Integrins control dendritic spine plasticity in hippocampal neurons through NMDA receptor and Ca2+/calmodulin-dependent protein kinase II-mediated actin reorganization. J Neurosci. 2006;26(6):1813–22. doi: 26/6/1813 [pii]; 10.1523/JNEUROSCI.4091-05.2006 16467530 PMC6793632

[24] Kerrisk ME, Cingolani LA, Koleske AJ. ECM receptors in neuronal structure, synaptic plasticity, and behavior. Prog Brain Res. 2014;214:101–31. 10.1016/B978-0-444-63486-3.00005-0 25410355 PMC4640673

[25] Bourgin C, Murai KK, Richter M, Pasquale EB. The EphA4 receptor regulates dendritic spine remodeling by affecting beta1-integrin signaling pathways. J Cell Biol. 2007;178(7):1295–307. 10.1083/jcb.200610139 17875741 PMC2064660

[26] Kawaguchi SY, Hirano T. Integrin alpha3beta1 suppresses long-term potentiation at inhibitory synapses on the cerebellar Purkinje neuron. Mol Cell Neurosci. 2006;31(3):416–26. 10.1016/j.mcn.2005.10.012 . 16307893

[27] Troca-Marin JA, Alves-Sampaio A, Tejedor FJ, Montesinos ML. Local translation of dendritic RhoA revealed by an improved synaptoneurosome preparation. Mol Cell Neurosci. 2010;43(3):308–14. 10.1016/j.mcn.2009.12.004 . 20035871

[28] Lee KJ, Kim H, Kim TS, Park SH, Rhyu IJ. Morphological analysis of spine shapes of Purkinje cell dendrites in the rat cerebellum using high-voltage electron microscopy. Neurosci Lett. 2004;359(1–2):21–4. 10.1016/j.neulet.2004.01.071 . 15050702

[29] Jones EV, Bernardinelli Y, Tse YC, Chierzi S, Wong TP, Murai KK. Astrocytes control glutamate receptor levels at developing synapses through SPARC-beta-integrin interactions. J Neurosci. 2011;31(11):4154–65. 10.1523/JNEUROSCI.4757-10.2011 . 21411656 PMC6623508

[30] Pozo K, Cingolani LA, Bassani S, Laurent F, Passafaro M, Goda Y. beta3 integrin interacts directly with GluA2 AMPA receptor subunit and regulates AMPA receptor expression in hippocampal neurons. Proc Natl Acad Sci U S A. 2012;109(4):1323–8. doi: 1113736109 [pii]; 10.1073/pnas.1113736109 22232691 PMC3268285

[31] Su J, Stenbjorn RS, Gorse K, Su K, Hauser KF, Ricard-Blum S, et al Target-derived matricryptins organize cerebellar synapse formation through alpha3beta1 integrins. Cell Rep. 2012;2(2):223–30. 10.1016/j.celrep.2012.07.001 22884367 PMC3432753

[32] Cingolani LA, Thalhammer A, Yu LM, Catalano M, Ramos T, Colicos MA, et al Activity-dependent regulation of synaptic AMPA receptor composition and abundance by beta3 integrins. Neuron. 2008;58(5):749–62. doi: S0896-6273(08)00337-1 [pii]; 10.1016/j.neuron.2008.04.011 18549786 PMC2446609

[33] Babayan AH, Kramar EA, Barrett RM, Jafari M, Haettig J, Chen LY, et al Integrin dynamics produce a delayed stage of long-term potentiation and memory consolidation. J Neurosci. 2012;32(37):12854–61. doi: 32/37/12854 [pii]; 10.1523/JNEUROSCI.2024-12.2012 22973009 PMC3752079

[34] Lai KO, Ip NY. Synapse development and plasticity: roles of ephrin/Eph receptor signaling. Curr Opin Neurobiol. 2009;19(3):275–83. 10.1016/j.conb.2009.04.009 . 19497733

[35] Hruska M, Dalva MB. Ephrin regulation of synapse formation, function and plasticity. Mol Cell Neurosci. 2012;50(1):35–44. 10.1016/j.mcn.2012.03.004 22449939 PMC3631567

[36] Murai KK, Pasquale EB. Eph receptors and ephrins in neuron-astrocyte communication at synapses. Glia. 2011;59(11):1567–78. 10.1002/glia.21226 . 21850709

[37] Filosa A, Paixao S, Honsek SD, Carmona MA, Becker L, Feddersen B, et al Neuron-glia communication via EphA4/ephrin-A3 modulates LTP through glial glutamate transport. Nat Neurosci. 2009;12(10):1285–92. 10.1038/nn.2394 19734893 PMC3922060

[38] Yu X, Wang G, Gilmore A, Yee AX, Li X, Xu T, et al Accelerated experience-dependent pruning of cortical synapses in ephrin-A2 knockout mice. Neuron. 2013;80(1):64–71. 10.1016/j.neuron.2013.07.014 24094103 PMC3792401

[39] Martone ME, Holash JA, Bayardo A, Pasquale EB, Ellisman MH. Immunolocalization of the receptor tyrosine kinase EphA4 in the adult rat central nervous system. Brain Res. 1997;771(2):238–50. . 9401744 10.1016/s0006-8993(97)00792-0

[40] Bi X, Lynch G, Zhou J, Gall CM. Polarized distribution of alpha5 integrin in dendrites of hippocampal and cortical neurons. J Comp Neurol. 2001;435(2):184–93. 11391640 10.1002/cne.1201

